# Unusual Etiology of an Ischemic Stroke: Total Thrombosis of the Right Internal Carotid Artery in a Case Report

**DOI:** 10.1002/ccr3.70193

**Published:** 2025-02-10

**Authors:** Wendlassida Martin Nacanabo, Taryètba André Arthur Seghda, Anna Tall/Thiam, Nobila Valentin Yameogo, André Koudnoaga Samadoulougou, Partice Zabsonre

**Affiliations:** ^1^ Cardiology Department Bogodogo University Hospital Center Ouagadougou Burkina Faso; ^2^ Cardiology Department Yalgado Ouedraogo University Hospital Center Ouagadougou Burkina Faso

**Keywords:** Burkina Faso, carotid artery, stroke, thrombosis

## Abstract

This case presents an unusual finding of total thrombosis of the right internal carotid artery complicated by a large patch of cerebral ischaemia in the right parieto‐temporo‐occipital and right capsulo‐lenticulo‐thalamic regions. The patient was managed mainly with medication, due to the lack of human and material resources required for any intervention.

## 1 Introduction

Ischaemic stroke is a serious cerebral pathology resulting from a sudden interruption of blood flow to a region of the brain, leading to neurological deficits that can be disabling [1]. The most common causes of ischaemic stroke include atherosclerosis of the cerebral arteries, cardiac embolism, and coagulation disorders [2]. However, some forms of ischaemic stroke may have atypical aetiologies that pose significant diagnostic challenges and require special attention. Total thrombosis of the internal carotid artery (ICA) is an unusual but critical cause of ischaemic stroke [3]. When the IC is completely blocked by thrombus, the risk of serious neurological complications is significantly increased, with potentially devastating consequences for brain function [4]. These cases are all the more instructive in that they add to our understanding of the rare aetiological mechanisms of ischaemic stroke and help to improve diagnostic and therapeutic approaches in similar situations. The case we present highlights a total thrombosis of the right ICA, a rare event that provides an opportunity to examine an uncommon etiology of ischaemic stroke at the Bogodogo University Hospital.

## 2 Case Presentation

### 2.1 History of the Disease

She was a 74‐year‐old housewife living in Ouagadougou, with age as a cardiovascular risk factor and a sedentary lifestyle as a thromboembolic risk factor. The patient had a history of neoplasia of the left breast. She was being monitored for hypokinetic dilated cardiomyopathy. She was consulted at the third hour for an onset of motor deficit plus speech impairment.

### 2.2 Physical Examination

The physical examination revealed proportional left hemiparesis, good hemodynamic function with blood pressure of 110/76 mmHg and saturation of 98% in room air. The rest of the physical examination was normal.

### 2.3 Additional Examinations

**FIGURE 1:**
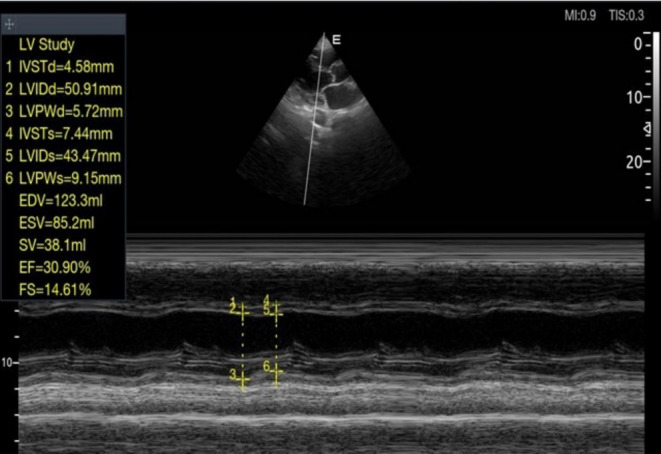
Doppler echocardiography in parasternal long‐axis section showing an alteration in the ejection fraction of the left ventricle.

**FIGURE 2:**
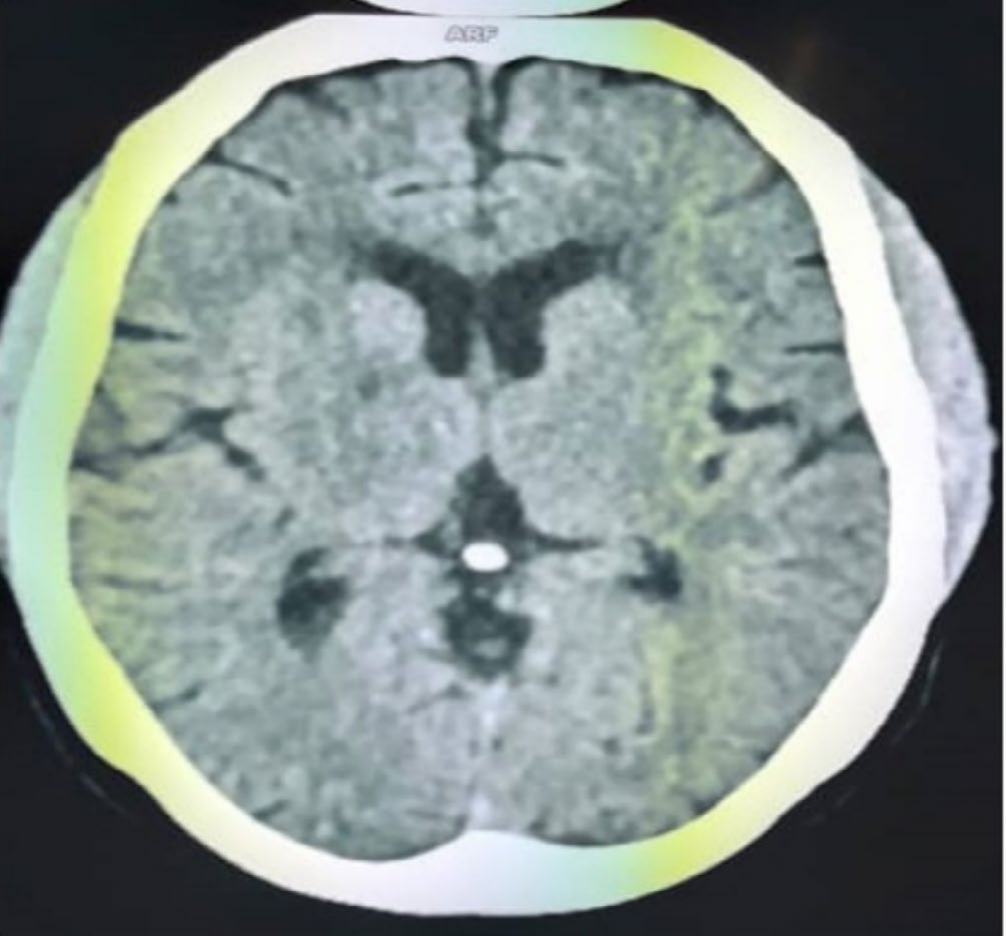
Cerebral tomodensitometry showing hypodensity of the posterior arm of the right internal capsule, diffuse hypodensity of the semi‐oval centres and of the periventricular white matter suggestive of an ischaemic stroke constituted in the territory of the right anterior choroid with cortico‐subcortical atrophy.

The electrocardiogram showed regular sinus tachycardia at 105 cycles per minute and atrial and left ventricular hypertrophy. On Doppler echocardiography, the left ventricular ejection fraction (LV EF) was 30% on the Teicholz scale, and left ventricular filling pressures were elevated, with pulmonary artery pressure elevated to 55 mmHg (Figure 1). Biological tests were normal. Cerebral computed tomography revealed small hypodensity of the posterior arm of the right internal capsule, diffuse hypodensity of the semioval centers, and periventricular white matter suggestive of ischemic stroke in the right anterior choroidal territory with cortico‐subcortical atrophy (Figure 2).

### 2.4 Primary Diagnosis

The diagnosis of ischemic stroke in the right anterior choroidal territory was made. Initial treatment consisted of Enoxaparin 0.0 mL/12 h, fluoxetine 20 mg (1 capsule/day) and Aspirin 100 mg (1 tablet/day).

### 2.5 Immediate Evolution

On his third day in the hospital, he developed proportional left hemiplegia and dysarthria.

### 2.6 New Explorations

**FIGURE 3:**
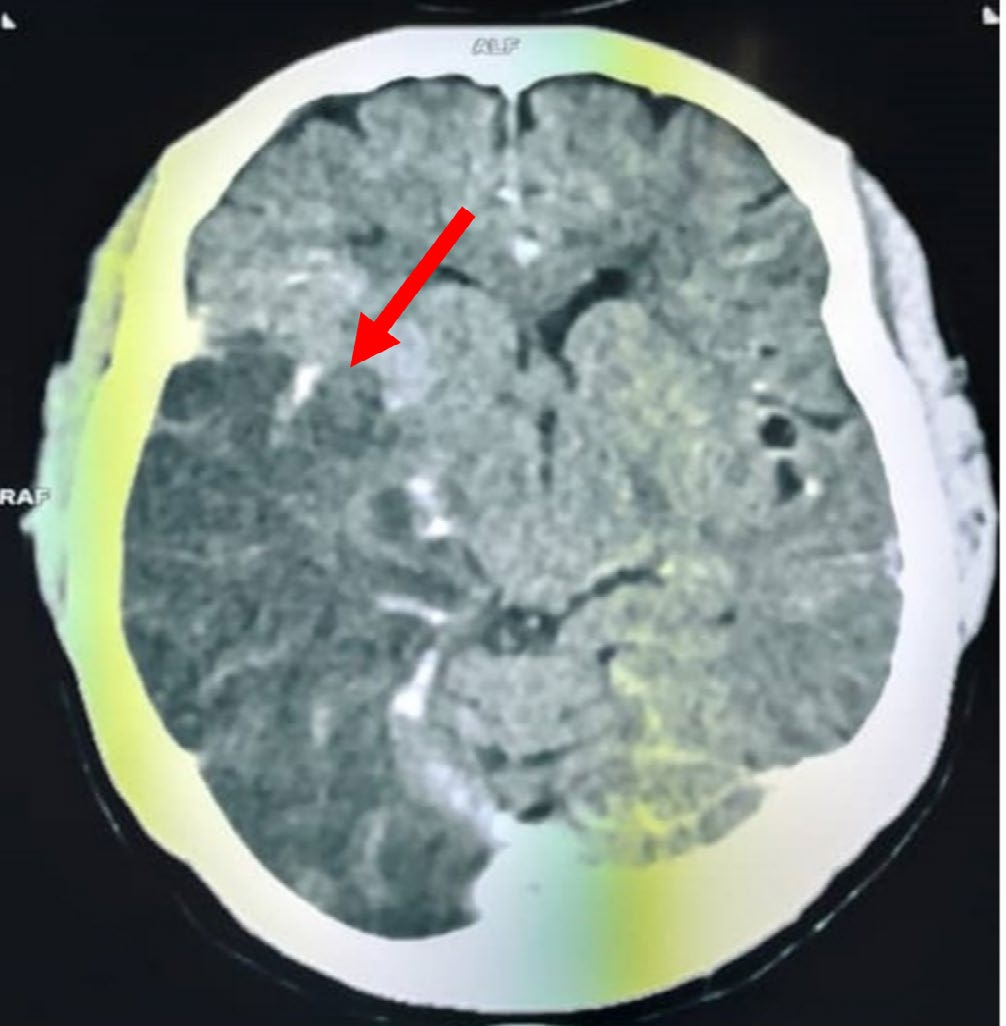
Cerebral CT scan showing a large area of hypodensity in the right parieto‐temporooccipital and right capsulo‐lenticulo‐thalamic regions of 24 Hounsfield units, responsible for a mass effect on the homolateral lateral ventricle, which is partially collapsed, and a deviation of the straight line of 4 mm (red arrow).

**FIGURE 4:**
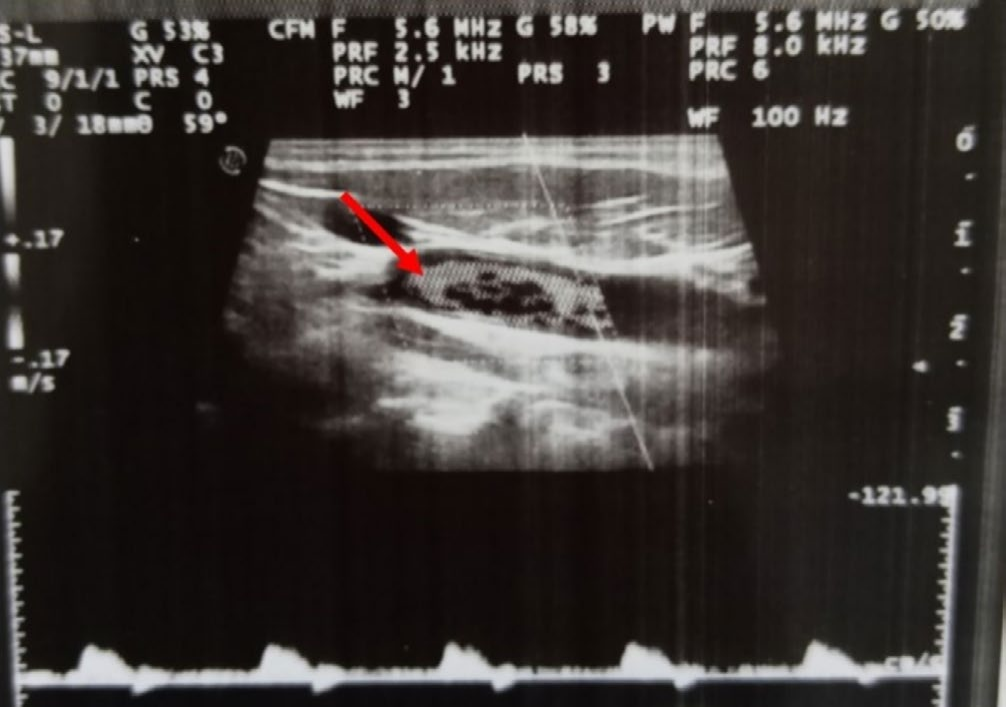
Doppler ultrasound of the supra‐aortic trunks (TSA) showing total thrombotic occlusion of the bulbar and post‐bulbar right internal carotid artery (red arrow).

**FIGURE 5:**
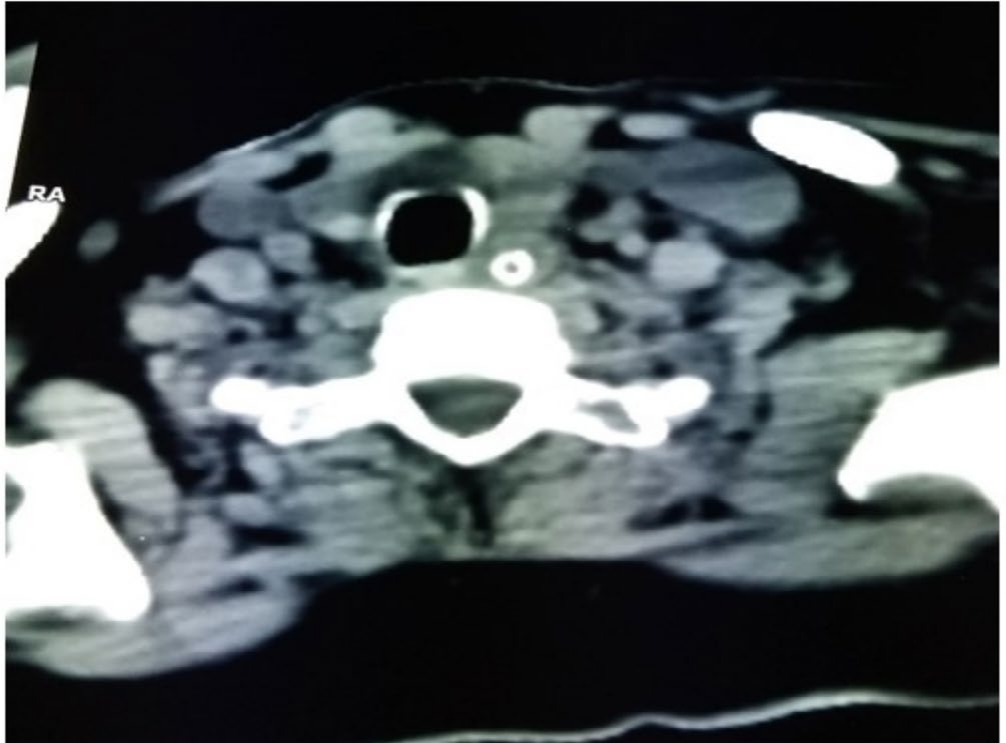
Angioscan of SAT showing right carotid atherosclerosis with an occlusive thrombus beginning at the bulb, extending to the internal carotid artery and into the supracavernous portion, without any associated vascular malformation.

A new CT scan of the brain revealed a large area of hypodensity in the left parieto‐temporooccipital and right capsulo‐lenticulo‐thalamic regions of 24 Hounsfield units, responsible for a mass effect on the homolateral lateral ventricle, which was partially collapsed, and a deviation of the straight line of 4 mm (Figure 3). There was no intra‐ or extra‐axial hemorrhage. Doppler ultrasound of the supra‐aortic trunks (SAT) showed a total thrombotic occlusion of the right internal bulbar and post‐bulbar carotid arteries (Figure 4). Angioscan of the SAT showed right carotid atherosclerosis with an occlusive thrombus beginning at the bulb, extending to the ICA and the supra‐cavernous portion, with no associated vascular malformation (Figure 5).

### 2.7 Diagnosis Selected

The diagnosis of ischaemic stroke was parieto‐temporo‐occipital and capsulolenticulo‐thalamic on the right, of atheromatous origin, with total thrombus of the right internal carotid artery on hypokinetic dilated cardiomyopathy with very reduced FeVG.

### 2.8 Treatment and Progress

Parenteral anticoagulation with Enoxaparin was continued for 10 days. The patient was discharged after 12 days in hospital on Aspirin 100 mg (1 tablet/day), Fluoxetine 20 mg (1 capsule/day) and Atorvastatin 40 mg (1 tablet/day).

## 3 Discussion

This case highlights several key points about the etiology, diagnosis, and management of this condition. Total thrombosis of the internal carotid artery is a rare cause of ischemic stroke [5]. Thrombosis of the ICA is a rare complication (0.2%–3%) of neck compression and is even less common in manual strangulation [6]. It can have serious consequences due to the significant interruption of blood flow to the cerebral territories supplied by this artery, leading to a stroke.

The diagnosis of ischemic stroke is made on the basis of clinical symptoms (motor deficit and speech impairment), imaging studies (cerebral tomography) and vascular investigations (Doppler ultrasound and angioscan of the SAT) [7]. In our patient's case, brain imaging revealed hypodense lesions consistent with ischaemic stroke and rapid progression of lesions, suggesting a dynamic course of thrombosis. The discovery of a total thrombosis of the right internal carotid artery, confirmed by Doppler ultrasound and angioscanner, was crucial for the diagnosis and management of ischaemic stroke. When a lesion of the fibrous cap occurs, contact of the lipid core with the luminal contents activates platelets and can activate the coagulation cascade, promoting the deposition of white and red blood cells on the plaque surface [8]. The absence of intra‐ or extra‐axial hemorrhage, together with the presence of carotid atherosclerosis, led to the suspicion that the thrombosis was of atheromatous origin, although the possibility of a platelet aggregate forming as a result of plaque fissuring could not be completely ruled out. For some authors, atherosclerosis is a systemic disease, so that medical treatment should be optimized in all patients with documented arteriosclerotic plaques of the carotid arteries [4]. In our patient the initial treatment included anticoagulation with Enoxaparin, an antiplatelet agent (Aspirin) and an antidepressant (fluoxetine), which are common choices in the management of ischaemic stroke to reduce the risk of new thrombus formation and improve clinical outcomes [9]. The patient's clinical course, with progressive worsening of neurological deficits and new brain lesions on CT scan, indicated that the thrombosis had resulted in extensive ischemia, requiring intensive treatment and close monitoring. Continued anticoagulation with Enoxaparin for 10 days, followed by aspirin, is in line with current recommendations for the treatment of ischemic stroke [10]. The patient was also placed on atorvastatin to treat atherosclerosis and reduce the risk of future events.

Carotid endarterectomy has been shown to be significantly more effective than the best medical treatment in patients with symptomatic ICA neurologically with severe stenosis (70% luminal narrowing) [11]. However, this type of surgery is not yet available in our context, and moreover, the indications must be based on the risks of each patient, since neurological and cardiac morbidity and mortality are significant risks. The lack of human and material resources makes it difficult to manage these lesions in our context.

## 4 Conclusion

In conclusion, this case of total thrombosis of the right ICA is a significant illustration of the range of rare aetiologies that can cause ischaemic stroke. This atypical finding highlights the importance of considering less common causes when clinical presentations do not fit the usual scenarios. The clinical course of this patient, despite appropriate treatment, highlights the severity of ischaemic stroke caused by total thrombosis of the right ICA and the importance of a multidisciplinary approach to the management of complications. Although surgery was necessary, it was managed solely with drugs, given the inadequacy of the technical resources available. The management of these rare forms of ischaemic stroke requires an individualized and often multidisciplinary approach to optimize clinical outcomes.

## Author Contributions

**Wendlassida Martin Nacanabo:** conceptualization, data curation, formal analysis, investigation, visualization, writing – original draft. **Taryètba André Arthur Seghda:** visualization, writing – review and editing. **Anna Tall/Thiam:** writing – original draft. **Nobila Valentin Yameogo:** writing – original draft. **André Koudnoaga Samadoulougou:** supervision. **Partice Zabsonre:** supervision.

## Consent

We have obtained the patient's consent for publication. Written informed consent was obtained from the patient to publish this report in accordance with the journal's patient consent policy.

## Data Availability

The data that support the findings of this study are available from the corresponding author upon reasonable request.
